# P-1860. Staphylococcus aureus nasal colonization and transmission is enhanced by concurrent influenza A virus upper respiratory infection in an infant mouse model

**DOI:** 10.1093/ofid/ofae631.2021

**Published:** 2025-01-29

**Authors:** Robert J Ulrich, Stacey Bartlett, Sabrina M Morales, Hedy Rocha, Mila B Ortigoza

**Affiliations:** NYU Grossman School of Medicine, New York, New York; NYU Grossman School of Medicine, New York, New York; NYU Grossman School of Medicine, New York, New York; NYU Grossman School of Medicine, New York, New York; NYU Grossman School of Medicine, New York, New York

## Abstract

**Background:**

*Staphylococcus aureus* asymptomatically colonizes the anterior nares of 20-30% of the population, and nasal carriage is a major risk factor for invasive *S. aureus* disease. Furthermore, rates of *S. aureus* nasal colonization are higher (40-50%) during the first eight weeks of life and colonizing strains can be transmitted among household contacts. Affecting the same niche, influenza A virus (IAV) is a common cause of viral upper respiratory infection (URI), and IAV predisposes the host to post-URI secondary *S. aureus* pneumonia. Despite the frequency of *S. aureus* and IAV in a shared anatomic site, the effects of IAV on *S. aureus* nasal colonization and transmission are understudied, and infant animal models that may better represent the younger high-colonization age group are not reported.

Infant mouse model

**Figure:**
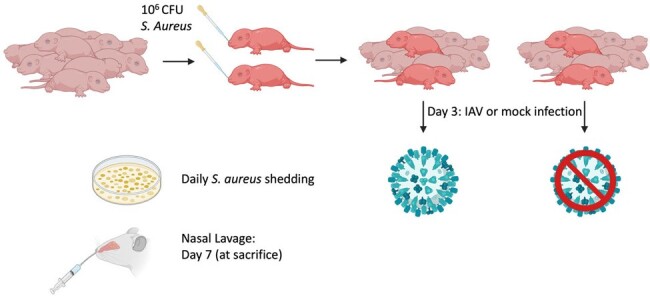

Two index pups from each litter were inoculated with S. aureus, and on day three the entire litter was either infected with IAV or mock (PBS) solution. S. aureus shedding is monitored daily and nasal lavage was performed.

**Methods:**

We established nasal colonization in infant B6 mice by inoculating 10^6^ colony forming units (CFU) of *S. aureus* MRSA strain JE2 and quantified nasal shedding for seven days on *S. aureus* chromogenic agar plates. For transmission experiments, we colonized two index pups per cage with *S. aureus* and infected the groups of pups with Influenza A virus (IAV) or mock (PBS) control on day three after *S. aureus* colonization. Nasal shedding of *S. aureus* was quantified daily and nasal lavage was performed at day seven.

**Results:**

Infant mice maintain *S. aureus* nasal colonization at seven days post-inoculation and they can transmit the colonizing strain to naive co-housed pups. IAV infection increases the *S. aureus* colonization burden 1000-fold by day 5 and the transmission to co-housed naïve pups by 63%.

**Conclusion:**

Influenza A virus infection increases nasal colonization burden of *S. aureus* and transmission to *S. aureus*-naive close contacts in an infant mouse model. We anticipate the model to be a starting point for the study of bacteria-virus interactions that contribute to *S. aureus* colonization in a high-risk population. Moreover, our work will allow for the study of both *S. aureus* and IAV mechanisms that contribute to *S. aureus* transmission amongst close contacts.

**Disclosures:**

All Authors: No reported disclosures

